# Venereal Disease in the Lower Animals*Reprinted from the *Veterinarian*, January, 1886.

**Published:** 1886-04

**Authors:** Thomas Walley

**Affiliations:** Principal of the Royal (Dick) Veterinary College


### Art. X.—VENEREAL DISEASE IN THE LOWER ANIMALS.*

BY PROF. THOMAS WALLEY, PRINCIPAL OF THE ROYAL (DICK) VETERINARY COLLEGE.

(Read before the Scottish Metropolitan Veterinary Medical Society).

In bringing before your notice the subject of "Venereal Disease in the Lower Animals," I may state at once that I have several objects in view. The first of these is to inquire as to what extent our domestic animals are liable to such affections. The second is as to whether we are justified, in the present state of our knowledge, in assuming that they suffer from conditions allied to those which are characteristic of syphilis in man. The third is to direct attention to a method of treating intractable venereal sores in the dog which is both sure and effectual.

In reference to the first of these questions, I may remind you that veterinary practitioners in this country are well acquainted with the fact that several of our domestic animals are sometimes affected with the disease known as *gonorrhœa* or, as it is termed in cows, "bull-burnt."

This specific form of urethritis, for such it is, occasionally makes its appearance in herds under such circumstances as to warrant the expression of an assumption that it is in such cases generated autogenetically.

I have myself seen it appear in herds in which neither the male nor the female members had ever been allowed to come in contact with other animals for procreative purposes, and I am well aware that other practitioners have seen it arise under identical conditions. In the matter of the dog, I have frequently had cases brought to me in which the owners of the animals have declared that they (the dogs) have never been in contact with any female for copulative or other purposes. When once the disease arises in the male its spread is not difficult to explain, and I have notes of some cases in a number of mares which came under the observation of one of my late

* Reprinted from the *Veterinarian*, January, 1886.

pupils, Mr. Barclay, of Dunfermline, and which were all traceable to infection from one stallion, who, it was subsequently shown, was the subject of the malady.

In France there exists a well-known form of venereal disease known as " La maladie du coit." Happily—at least, so far as I know—the equine species in this country is exempted therefrom. Neither have I ever seen any such form of disease in the sheep or in the pig.

As to how far gonorrhœa may be looked upon as a specific disease, I may first direct your attention to the fact that the literal meaning of the word used to designate this class of affection is totally out of accord with its proper application. It signifies an excessive flow of semen—a condition sometimes observed in young vigorous dogs when excited by the near proximity of œstral bitches, and which, owing to the fact that it is accompanied by tumefaction of the sheath and swelling of the testes, is confounded with the affection usually termed gonorrhœa. The use of the term is, however, warranted by custom, and I do not feel disposed to cavil at its application to the form of disease under consideration.

As to the specificity of gonorrhœa, I may remind you that the researches of those engaged in inquiries as to the nature of the disease in the human subject have not resulted in any definite issue. True, the statement has been made that a micrococcus has been detected in the discharges of gonorrheal urethritis, and in those of gonorrheal ophthalmia, identical in its characters from both sources; but there are those who assert that in urine which has undergone fermentative changes a micrococcus, recognized as *M. urinæ*, is always present; further, inquirers—MM. Lepine and G. Roux—have recently drawn attention to the fact that when cultivations of this now well-known micrococcus are introduced into the urethral canal of male animals, and retained there for a short time, a specific urethritis is established, which extends to the bladder and kidneys, and produces death, with reproduction in large numbers of the micrococcus in the urinary products; and of still greater importance is the further statement that healthy females confined in cages with these experimentally-infected animals become the subjects of identical conditions, and die with similar urethral, cystic, and renal lesions well developed.

Now, if these altered micrococci were the actual cause of gonorrhœa, we should expect that, in old-standing cases, the bladder and kidneys would become diseased; such, however, is never observed—at least in my experience. That specific urethritis in animals is identical with that of man is proved by the fact that in dogs we not infrequently see well-marked gonorrheal conjunctivitis produced by contact of the urethral discharge with the conjunctivæ. The infection is conveyed by the animal licking its penis, and subsequently one of the hind feet, from which the virus is passed to the conjunctiva in the act of scratching. Mr. Alex. Gray informs me that some years ago he saw a monkey—which had contracted the disease from handling contaminated lint thrown away by a sailor on board ship—with gonorrheal conjunctivitis, the infection having been conveyed to the membrane by the creature's own hand.

So far, then, we see that gonorrhœa affects the horse, the ox, the dog and the monkey; that in these animals its course is similar to that which it follows in man. But what about our second query: "Do the domestic animals suffer from syphilis?"

The great characteristics of syphilis are—firstly, that it is a disease running a chronic course; secondly, that its most prominent lesions are indolent ulceration of mucous membranes and skin, with chronic enlargement and induration of lymphatic glands, and ulceration of the skin covering these glands.

Do we meet with such lesions in veterinary practice? Most certainly yes—in the dog, at least. Are they of a syphilitic nature and origin? I will answer this question by expressing the opinion that they are of that nature, and I think I am warranted in so doing by the characters presented by these lesions and by their course.

I am quite aware that objection may be taken to my conclusions on the ground that the chronic lesions seen in animals are only results—or *secondary symptoms*, if you like—of gonorrhœa. So used pathologists to consider the lesions of syphilis in man, but they have now come to look upon them as being totally different, both in nature and in cause.

I know it has been said that the *chancre* of the dog differs materially from that seen in man, in the fact that its edges and base are less indurated. Granted this to be the case, may not, I ask, any slight difference in character be due to differences in structure (histologically) in the membranes in the two animals?

A chancre makes its appearance on the penis of the dog; it gradually extends its bounds; other chancres form, and in some instances the primary and secondary sores coalesce; from the surface of these sores a puriform fluid, ichorous and infective in its character, is discharged; this fluid sets up a degenerative inflammation in the skin of the thighs, the abdomen, the chin, and in fact in all parts with which it comes in contact. These sores do not yield to the local application of caustics, and in many cases are affected only to a limited extent by the internal administration of potassic iodide and mercury, unless, indeed, these drugs are pushed to a dangerous extent.

In the course of time the dog begins to arch his back, and the motion of the hind legs becomes stiff and the gait straddling. By these signs the attention of the practitioner is directed to the inside of the thigh, and he there detects a *bubo* identical in its character with that seen in man, and, like it, ultimately associated with chronic ulceration of the skin covering it. If these lesions are not syphilitic, what are they? I leave those who deny that the disease exists in the lower animals to answer the question.

In reference to the treatment of those important lesions *bubo* and *chancre*, I may observe that the former is much more easily dealt with than the latter. In my experience, the free inunction of iodine ointment, with the application of nitrate of silver to ulcerated surfaces when such are present, quickly produces resolution, but in obstinate cases I should advise iodine irrigation or extirpation of the tumor; no harm could result from the adoption of the latter course, and seeing that there is great danger of the gland becoming a centre of infection to the system, it would be the wisest course to adopt.

In the treatment of chancrous ulceration of the penis we have an infallible remedy, if I be permitted to so designate it, in castration. I am perfectly well aware that some of my scientific friends will utter an exclamation of surprise and horror when this statement meets their eyes, but one fact is worth a dozen theories, and however unscientific and unsurgical the operation may at first sight appear, it is all the same an absolutely effectual and perfectly safe cure. In the case of valuable stud dogs the removal of the testicles would, I need scarcely say, be a matter of grave importance, as its adoption would mean annihilation of the procreative function ; but in dealing with animals in which their use for stud purposes is only of secondary importance, the operation should unhesitatingly be performed if other remedies fail in affecting a cure. The first occasion on which I adopted the treatment in practice was in October, 1873, at which time I had under my care a terrier of nondescript breed (much prized as a companion by his owner) suffering from venereal sores on the penis and on the skin of the abdomen and thighs. After giving the usual constitutional remedies, as potassic iodide and mercury, and the usual local applications, as nitrate of silver and sulphate of copper, a lengthened trial, I determined upon trying the effects of castration. My reason for performing the operation was that I observed whenever I manipulated the animal chordee became very marked, and this was followed by extreme vascularity of the penis and particularly of the tissues involved in the ulcerative process. In my own mind I came to the conclusion that if priapism were prevented this periodical hyperæmia would be done away with and rest insured to the cells of the diseased parts.

The operation was performed, the effects of it surpassed my most sanguine expectations, and the dog was quickly discharged cured.

In the early part of the present year I was asked to examine a retriever dog, in whom an obstinate eruption was presented on the skin covering the chin and around the eyes. I was informed that the animal had been under the care of another practitioner for some time, but that the treatment adopted had been unsuccessful. As my attention was only directed to this eruption, I did not think of looking further for the probable cause, though I was particularly struck by the peculiar character of the sores, and unhesitatingly gave the opinion that they were of a specific nature. By the application of napthol ointment, an agent, I may observe, of great value in some forms of skin disease, and the administration of mercury, the sores rapidly improved, and in fact were nearly healed, but several weeks subsequently the owner of the dog noticing an eruption of the skin of the abdomen, again brought the dog to me for examination. One glance at this eruption satisfied me as to its source and explained the origin of the sores around the eyes and on the chin ; and, on extruding the penis, my suspicions were confirmed, the organ presenting on its external surface several well-marked venereal sores. These sores were treated, successively, with all the known topical remedies, not even excluding iodoform; and simultaneously, constitutional treatment was employed, but all to no purpose, and in the end the penis presented a most loathsome and horrible appearance. With the consent of the owner, castration was performed. The good effects of the operation was as satisfactory as in the first case.

I may remark that I have been informed, within the last few days, by one of my pupils, Mr. Carruthers, of Wigton, Cumberland, that during the past summer an equally successful result was obtained by the adoption of this method of treatment in the case of a collie which had been the subject of venereal sores for a considerable period.

I have often felt that this treatment, heroic though it be, might be had recourse to with benefit in the human subject. I know that sentiment would oppose itself to the suggestion, but I would ask is it not better that a man who has been unfortunate enough to contract this loathsome disease should be deprived of the means of infecting others, and be himself restored to at least a moderate degree of capacity for enjoyment of the pleasures of life than that he should be allowed to drag out a miserable existence and remain a probable means of disseminating his infirmity ?

